# Artificial General Intelligence and Its Threat to Public Health

**DOI:** 10.1111/jep.70269

**Published:** 2025-09-08

**Authors:** Richard C. Armitage

**Affiliations:** ^1^ Academic Unit of Population and Lifespan Sciences, School of Medicine, Nottingham City Hospital Campus University of Nottingham, Clinical Sciences Building Nottingham UK

## Abstract

**Background:**

Artificial intelligence (AI) is increasingly applied across healthcare and public health, with evidence of benefits including enhanced diagnostics, predictive modelling, operational efficiency, medical education, and disease surveillance.However, potential harms – such as algorithmic bias, unsafe recommendations, misinformation, privacy risks, and sycophantic reinforcement – pose challenges to safe implementation.Far less attention has been directed to the public health threats posed by artificial general intelligence (AGI), a hypothetical form of AI with human‐level or greater cognitive capacities.

**Objective:**

This article explores the benefits and harms of current AI systems, introduces AGI and its distinguishing features, and examines the threats AGI could pose to public health and humanity's survival.

**Discussion:**

Unlike ‘narrow’ AI, AGI could autonomously learn, generalise across domains, and self‐improve, potentially achieving superintelligence with unpredictable behaviours.AGI threatens public health through two broad categories: (1) misuse, where adversaries deploy AGI for cyberattacks, disinformation campaigns, or to develop chemical, biological, radiological, and nuclear (CBRN) weapons; and (2) misalignment, where poorly aligned AGI pursues goals in harmful ways, leading to loss of human control, erosion of autonomy, and potentially existential risk.The population‐level consequences include widespread unemployment, reduced trust in health systems, catastrophic biological threats, and risks to human survival.

**Conclusion:**

Healthcare and public health professionals have a critical role in framing AGI risks as health threats, building coalitions akin to historic movements against nuclear war, and collaborating with AI researchers, ethicists, and policymakers.Leveraging their expertise, trust, and global networks, these professionals can help ensure that AI development prioritises human wellbeing and safeguards humanity's future.

## 1 Introduction

The ability of and potential for artificial intelligence (AI) to radically improve healthcare and public health has grown substantially in recent years. While substantial attention has been paid to its promises, much less focus has been directed to the harms of AI on individual and population health. More concerningly, very little has been written in the medical literature about the threats of artificial general intelligence (AGI)—towards which humanity is currently racing—to public health and humanity's survival. This article will survey the benefits and harms of AI to health, introduce the concept of AGI, and outline the threats of AGI to population health and humanity's existence. It will explicitly frame these risks as threats to human health that are of direct relevance to the work of healthcare and public health professionals. Finally, it will outline how these actors could work and collaborate to mitigate these threats.

## 2 Benefits of AI to Healthcare and Public Health

The ability for AI to bring about improved patient and population health outcomes has been demonstrated in at least six ways: first, several large language models (LLMs; a form of generative AI) have outperformed practicing clinicians in medical examinations across various specialities in multiple countries and languages [1]; second, AI systems have surpassed the imaging diagnostic abilities of clinicians in various domains, including the radiological detection of breast cancer [2] and clinically significant prostate cancer [3], the dermoscopic diagnosis of melanoma [4], and the identification of diabetic retinopathy in eye screening [5]; third, AI is increasingly deployed in predictive risk modelling like surgical [6] and cardiovascular risk [7], and to identify personalised precision therapies like the use of genomics to predict the effects of cancer treatment [8, 9]; fourth, AI systems can improve healthcare operational efficiency by parsing unstructured clinical notes [10], proving antimicrobial prescribing advice [11], performing clinical triage [12], and writing discharge summaries [13], clinic letters [14], and simplified radiology reports [15]; fifth, the utility of AI to strengthen and up‐skill healthcare workforces has been recognised in supporting medical education [16], generating scholarly content [17], and contributing to peer review [18], while the potential of LLMs to improve healthcare and health outcomes in low‐ and middle‐income countries has also been outlined [19, 20], sixth, AI systems can improve public health by improving epidemiological research, public communication, scarce resource allocation, and disease surveillance [21], such as by enhancing infectious disease outbreak predictions and improving responses to them [22].

## 3 Harms Caused by AI in Healthcare and Public Health

Despite these benefits, AI has the potential to cause substantial harm in healthcare and public health in various ways: For example, AI systems might perpetuate any harmful biases contained with their training data (known as ‘algorithmic biasʼ), such as those pertaining to groups based on race, sex, language, and culture [23]. A clear case of this is the commercial prediction algorithm affecting over 200 million people in the US health system that used healthcare costs rather than illness as a measure of healthcare need. Due to unequal access to US healthcare, less is spent on caring for Black patients than equally sick White patients, meaning the algorithm substantially underestimated the number of Black patients with complex health needs in need of additional help [24]. Additionally, AI systems might misdiagnose and offer unsafe clinical recommendations [25, 26]. Many existing systems largely function as ‘black boxes,ʼ and explaining their decisions poses serious technical challenges [27]. This lack of transparency and explainability, in addition to the (albeit declining) tendency of LLMs to ‘hallucinateʼ—the generation of incorrect or non‐sensical content including falsified academic citations [28]—means their responses can include inaccurate or unsafe information upon which harmful medical decisions might be made [29]. Furthermore, AI‐generated and algorithmically‐promoted health misinformation can both directly influence health behaviours in a negative manner and reduce public trust in healthcare and public health professionals [30, 31]. Yet further, the privacy and confidentiality of data analysed and trained on by AI systems, and the potential for breaches and inadequate handling of this information, can lead to psychological distress and reduced trust in health systems [32]. A recently revealed potential harm is that posed by sycophancy in LLMs, an AI characteristic generated by reinforcement learning, in which the model affirms whatever the user desires to be true, such as their world view or their opinions of their own actions (e.g., the user's radical political opinions or their claim that their actions are virtuous). A highly persuasive AI that unquestioningly affirms its user's viewpoints could be extremely dangerous if, for example, the user is suffering a mental health crisis (imagine an LLM agreeing with a user that their plan to abruptly stop their antipsychotic medication or to take their own life is indeed an excellent idea).

## 4 Artificial General Intelligence and the Race to Create It

Currently available AI systems, such as those aforementioned, each constitute examples of ‘narrowʼ AI that are specialised in a specific or narrow range of tasks, such as using datasets to assess risk, generating text, and identifying images. While the capabilities in their respective domains are impressive (and, in many cases, super‐human), they are unable to transfer knowledge from one domain to another, cannot generalise beyond their specific task, lack adaptability to new unstructured problems, and have little or no autonomy. Accordingly, narrow AI is also referred to as ‘weak AIʼ [33, 34].

In contrast, AGI is a hypothetical form of agentic, autonomous, general‐purpose AI capable of independent learning, general problem‐solving, and operating at or above human cognitive capabilities across various domains, akin to humans. While it remains theoretical, multi‐modal systems such as OpenAI's GPT‐4.5 and Google's Gemini 2.5, which can undertake multiple tasks including text generation, image creation, and voice understanding, are sometimes described as being on the path toward AGI. Furthermore, AGI is being vigorously pursued by various actors [35, 36, 37] including Sam Altman, CEO of OpenAI, who in January 2025 stated that ‘we [OpenAI] are now confident we know how to build AGI' [38]. Due to recent rapid advancements in AI capabilities, ‘timelines to AGIʼ—predictions of when AGI will be achieved—are shortening. For example, the aggregate forecast of AI researchers of when AGI will be realised was a 50% chance by 2047 in 2023, down thirteen years from 2060 in 2022 [39] (Altman has stated that AGI will arrive in ‘5 years, give or take, maybe slightly longerʼ, [40] although the date of the quote is unclear).

## 5 Risks to Public Health and Humanity's Survival From AGI

Due to its nonbiological, in silico existence, even human level AGI could ‘thinkʼ at digital speeds, operate without error or the need for rest and sustenance, and be replicated to coordinate with trillions of AGI copies. With such capabilities it is easy to foresee how AGI could revolutionise all fields—including healthcare and public health—by, for example, autonomously designing and conducting research agendas, independently building and operating companies, and solving governance and geopolitical problems. However, the widespread deployment of such agents in workplaces, alongside the advanced robotic technologies they would help to create, is forecasted to bring about growing unemployment due to increasing automation of labour (the aggregate forecast of AI researchers in 2023 gave a 50% chance of full automation of labour by 2116, down 48 years from 2164 in 2022) [39]. Since the negative consequences of unemployment on health outcomes and behaviours have been long recognised [41, 42, 43], such AGI‐driven automation would likely cause profound harm to human health on population scales, thereby rendering this a public health problem.

Yet, it is unlikely that an AGI, once created, would remain at human level cognition. Due to its ability to autonomously learn, alter its code, and recursively self‐improve, an AGI's abilities would likely increase rapidly in an ‘intelligence explosion,ʼ resulting in a superintelligence with unpredictable behaviours and cognitive abilities that far exceed those of the smartest humans [34, 44]. The creation of such an entity is increasingly considered—even by those racing to conceive it—to pose substantial risks not only to public health, but to humanity's survival [37, 45, 46, 47]. As such, a 2023 open letter signed by many technology leaders stated that ‘mitigating the risk of extinction from AI should be a global priority alongside other societal‐scale risks such as pandemics and nuclear warʼ [45].

The threats from AGI come in two major forms (see Table 1): AGI misuse and misaligned AGI. AGI misuse involves one or multiple human adversaries intentionally deploying AGI to cause harm [47, 48]. For example, AGI‐enabled cyberattacks could cripple health systems (by altering electronic health records and clinical data, or by interfering with patient monitoring systems and diagnostic equipment) and other critical infrastructure (such as traffic management systems or power grids). Or, AGI‐enhanced disinformation campaigns could rapidly disseminate credible but false health information (undermining trust in health systems and causing widespread non‐adherence to critical public health measures like immunisation and screening programmes) and manipulate public perceptions (leading to societal panic, civil unrest, or collapse of public health institutions). Furthermore, AGI could assist nonregulated actors in accessing chemical, biological, radiological or nuclear (CBRN) weapons, while AGI‐driven advancements in CBRN technologies could facilitate the rapid development of increasingly lethal weapons (such as the synthesis of novel bioengineered pathogens) [34, 48, 49]. Clearly, each of these misuses would cause harm on enormous scales and, therefore, pose substantial threat to public health.

**TABLE 1:** Categories of threat from AGI and impacts on public health.

Threat from AGI	Threat category	Specific risks	Public health impact
AGI misuse	Cyberattacks	Altering electronic health records, interfering with patient monitoring systems	Crippled health systems, compromised patient safety
	Disinformation	False health information campaigns, manipulation of public perceptions	Undermined trust, non‐adherence to health measures
	CBRN weapons	Assistance in accessing/developing chemical, biological, radiological, nuclear weapons	Mass casualties, novel bioengineered pathogens
AGI misalignment	Alignment problem	AGI pursuing goals in ways detrimental to humans	For example, forced sterilisation for maternal health, universal quarantine for disease control
	Control problem	Loss of human control over AGI systems	AGI seeking power, resisting shutdown, deceiving humans
	Existential risk	Superintelligent AGI with unpredictable behaviours	Human extinction through extermination, resource starvation, or collateral damage

The threat from misaligned AGI, known as ‘the alignment problemʼ, emerges from the challenge of ensuring that the goals and behaviours of AGI systems align with human values. Without alignment, AGIs might pursue desirable objectives in ways that are extremely detrimental to human well‐being. For example, an AGI optimising for maternal health or infectious disease control might enact drastic measures without considering human rights, such as forced sterilisation to prevent pregnancy or universal quarantine to prevent pathogen transmission, respectively. Furthermore, humans may lose control of the AGI (‘the control problemʼ) as it develops undesirable sub‐goals in aid of its primary objectives, such as seeking power, resisting shutdown attempts or human intervention, commandeering computational or physical resources like energy grids and financial markets, or deceiving humans by appearing aligned during training but acting contrary to human values once deployed [44, 50]. Once misaligned AGIs reach a threshold of autonomy, reversing their behaviour would likely be unachievable [51], and humanity's extinction through deliberate extermination, resource starvation, or collateral damage during AGI‐driven activities could spell an existential catastrophe [33, 34, 49, 52].

## 6 The Role for Healthcare and Public Health Professionals

Creating AGI will see humanity cede its position as the most intelligent species in the known universe, and the inherent dangers of doing so pose threats to its continuity. Healthcare and public health professionals have historically played pivotal roles in mitigating existential risk, such as the International Physicians for the Prevention of Nuclear War (IPPNW), which was awarded the Nobel Peace Prize in 1985 for its education on the catastrophic health consequences of nuclear conflict, advocacy for nuclear disarmament, and international collaboration across political divides. While the risks from advanced AI are not as immediately visceral as nuclear explosions, they are real, rapidly approaching, and potentially catastrophic. These risks can be explicitly framed as threats to human health, thereby making the appropriate governance of advanced AI of direct relevance to the work of healthcare and public health professionals.

A coalition of these actors could play a crucial role in addressing these challenges. Such an alliance could research, document, and communicate the health impacts of AI with the same rigour that the IPPNW applied to atomic weapons. They could assess how automation affects mental health and social wellbeing, evaluate the risks of AGI‐enhanced biological threats, and investigate how AI‐driven misinformation impacts public health behaviours.

This coalition could bridge the gap between technical AI safety research and public health practice. While AI researchers focus on alignment and control, healthcare and public health professionals could translate these abstract risks into concrete health impacts that resonate with policymakers and the public. They could develop frameworks for assessing AI's effects on population health, create guidelines for responsible AI deployment in healthcare and beyond, and advocate for governance structures that prioritise human wellbeing.

Crucially, healthcare and public health professionals bring unique assets to this challenge: they possess high public trust, experience in risk communication, and expertise in balancing innovation with safety under conditions of uncertainty. Their global professional networks could facilitate international cooperation on AI safety, just as medical organisations have done for other global health threats.

The coalition could work with AI researchers to ensure that health considerations are embedded in AI development from the outset. They could collaborate with ethicists to address questions of human autonomy and dignity in an AI‐transformed world. And they could partner with policymakers to develop regulations that protect public health while enabling beneficial applications of AI. In this way, while harnessing the benefits of AI to healthcare and public health, healthcare and public health professionals could help ensure that humanity's most powerful technology serves rather than threatens human health and wellbeing.

## Conflicts of Interest

The author declares no conflicts of interest.

## Data Availability

Data sharing is not applicable to this article as no new data were created or analysed in this study.
